# Amateur Photography

**Published:** 1889-02

**Authors:** 


### AMATEUR PHOTOGRAPHY.

Great impetus has been given to amateur photography recently by the general introduction of a new developing agent, which greatly simplifies the impor ant and interesting process by which the latent images on the gelatine dry plates are brought to light. It is known as hydroquinone, and it is greatly to its advantage that it is neither patented nor in the slightest degree a secret composition. The chemical symbols of hydroquinone (or hydrochinon) are $C^6 H^4 (O H)^2$ and it is a product of dry distillation of kinic acid which is found in cinchona bark. The hydroquinone comes in colorless crystals, easily soluble in water, and may be purchased of any of the photographic stock dealers. A few stock dealers and plate makers have prepared a developer made of hydroquinone and put up in eight and sixteen ounce bottles, the larger sizes selling for from fifty to sixty cents each. This is the perfect one-solution developer.

The amateur, after retiring to his dark room, closing the door and producing his ruby light, has only to flood his plate in a tray with the solution from the bottle. He can do this in a careless and haphazard manner, for the image does not flash up instantly, and he need not flood the plate with the clean sweep so necessary with other developers, nor need he look carefully for the little air bubbles so annoying to amateurs and disastrous to plates under pyrogallic development. There is so little probability of a failure that it is a positive pleasure to develop plates with this new agent, and its advantages are manifold. It does not stain the fingers or the plates as the pyro developer did, and with it a black plate can always be obtained. Furthermore, there is less danger of plates filling when the plates are developed in warm weather if hydroquinone is used.

The new agent is equally good for developing paper negatives or positives, and gives a clear picture when used to develop lantern slides for which neutral oxalate developer has heretofore been used exclusively. There is nothing to do but to leave the plate in the solution and await developments. No adding a little more potash or a little more pyro as defects in the process become apparent as under the old plan, and no soaking of plates in clear water to arrest too rapid development; but chief of all the advantages of hydroquinone is the absence of all danger of fogging the plate except by the action of white light. If the hydroquinone is properly prepared chemical fog cannot appear.

Demonstrators of the use of the new developer will make five successive exposures, timing each one differently—undertiming two, overtiming two and having one normally timed. Then place the five plates in one tray, cover them with hydroquinone, and by removing them at precisely the right moment, produce five negatives exactly alike. This is what the average amateur has needed for years; something to correct his defects of exposure ; something which can be relied upon to produce good results where his judgment of the light has been bad, and hydroquinone comes nearer to doing it than anything hitherto produced.

With pyrogallic acid careless amateurs have been frequently afflicted with weak plates, with yellow plates and with plates obscured by fog or peforated with pinholes, and the careless amateur has frequently been known to other amateurs at large by the fact that his finger nails were always tinged with brown stain. Hydroquinone has accomplished a reform in all these, and its cleanliness and simplicity have brought back to the ranks of the amateur photographers many young and old people who were led to give it up by the vexatious failures and the chemical manipulations necessary under old methods. Next to the introduction of dry plates the discovery that hydroquinone was a developing agent is probably the most important epoch in the art of photography. Amateurs are not alone in adopting it, for it is in daily use in thousands of galleries in this land. It is now being made in several establishments in this country, so that photographers are not dependent upon foreigners for it as they are for pyrogallic acid.

